# Impact of Flow Rate, Collection Devices, and Extraction Methods on Tear Concentrations Following Oral Administration of Doxycycline in Dogs and Cats

**DOI:** 10.1089/jop.2018.0008

**Published:** 2018-07-01

**Authors:** Lionel Sebbag, Lucas Showman, Emily M. McDowell, Ann Perera, Jonathan P. Mochel

**Affiliations:** ^1^ Department of Biomedical Sciences, Iowa State University, College of Veterinary Medicine, Ames, Iowa.; ^2^ Department of Veterinary Clinical Sciences, Iowa State University, College of Veterinary Medicine, Ames, Iowa.; ^3^ W.M. Keck Metabolomics Research Laboratory, Iowa State University, Ames, Iowa.; ^4^ Lloyd Veterinary Medical Center, Iowa State University, College of Veterinary Medicine, Ames, Iowa.

**Keywords:** pharmacokinetics, doxycycline, oral administration, tear film, canine, feline

## Abstract

***Purpose:*** Compare the precision of doxycycline quantification in tear fluid collected with either Schirmer strips or polyvinyl acetal (PVA) sponges following oral drug administration.

***Methods:*** Three dogs and 3 cats were administered doxycycline orally at a dose of 4.2–5 mg/kg every 12 h for 6 consecutive days. At day 5 and 6, blood and tear fluid were sampled to capture doxycycline trough and maximal concentrations. Tear fluid was collected 3 times (spaced 10 min apart) at each session with the absorbent material placed in the lower conjunctival fornix until the 20-mm mark was reached (Schirmer strip, one eye) or for 1 min (PVA sponge, other eye). Tear extraction was performed with either centrifugation or elution in methanol. Doxycycline concentrations were measured with liquid chromatography–mass spectrometry. Low (100 ng/mL) and high (1,000 ng/mL) tear concentrations measured *in vivo* were spiked into each absorbent material *in vitro* to evaluate percentage drug recovery.

***Results:*** After oral administration of doxycycline, the drug reached the tear compartment at concentrations of 45.1–900.7 ng/mL in cats and 45.4–632.0 ng/mL in dogs, representing a tear-to-serum ratio of 12% and 16%, respectively. Doxycycline tear concentrations were significantly more precise when tear collection was performed with Schirmer strips rather than PVA sponges (*P* = 0.007), but were not correlated with tear flow rate. *In vitro* doxycycline recovery was poor to moderate (<75%).

***Conclusions:*** Schirmer strips represent a good option for lacrimal doxycycline quantification, although the collection and subsequent extraction have to be optimized to improve drug recovery.

## Introduction

Systemically administered medications are used to manage a variety of ocular surface diseases across species, for instance oral cetirizine for allergic conjunctivitis in humans,^1^ famciclovir for herpetic keratitis in cats,^2^ and doxycycline for keratomalacia in horses,^3^ among others. Although promising, the prerequisite for such therapies is a deep understanding of the drug pharmacokinetics–pharmacodynamics at the ocular surface,^4^ a process that first requires accurate quantification of the drug levels in the tear fluid. Indeed, under- or overestimating tear concentrations could cause a drug to be given at an inappropriate dose and/or frequency, thereby affecting its efficacy, increasing the risk for drug toxicity, or development of acquired resistance (for anti-infective drugs).^5,6^ However, accurate quantification of drug in tears is complicated by the lack of standardized method to collect and analyze this biological fluid.^7–10^ This is exemplified by doxycycline, a broad-spectrum antibiotic that has been shown to reach the tear compartment in various species: After oral administration, doxycycline tear concentrations were relatively high in horses (1,810–9,830 ng/mL),^11^ lower in cats (100–110 ng/mL),^12^ much lower in dogs (0.04–5.73 ng/mL),^13^ and nonexistent in humans.^14^ The subtle species differences in plasma protein binding of doxycycline^15^ cannot solely explain the large discrepancies in the observed tear concentrations; rather, it is likely that the concentrations obtained have been skewed by an unrecognized impact from the tear collection process, as is the case for tear protein content^16^ and other analytes like glucose,^17^ and warrants further investigation.

Direct tear collection with capillary tubes, although commonly used in clinical research because the sample obtained is readily available for analysis,^8^ presents several drawbacks that limit its use for drug quantification. In particular, the volume of tear fluid retrieved is generally low, thus rendering some samples insufficient for analysis^1^ unless previous stimulation or topical instillation of saline is performed.^18^ In addition, the drug recovery and the accuracy of analysis are lower than other methods, as shown by Small et al. who noted that capillary-collected samples underestimated ofloxacin concentrations in rabbit tears and resulted in greater variability when compared with Schirmer strips and surgical sponges.^19^

Indirect tear collection with Schirmer strips and ophthalmic sponges have been successfully used to quantify lacrimal drug levels in several species.^2,13,19,20^ These collection methods are easy to use, safe, and provide quantities of tear fluid adequate for most bioanalytical procedures.^7,21,22^ However, Schirmer strips and ophthalmic sponges can also cause reflex tearing due to ocular irritation and therefore affect the detection of the investigated tear component (eg, drug concentration).^21,22^ Standardization of the collection methods and controlling tear flow rate may hinder these physiological changes and improve the repeatability of the analysis.

The purpose of our study is to compare the repeatability (ie, precision) of drug quantification on the ocular surface from tears collected with Schirmer strips or ophthalmic sponges. We also aim to investigate the impact of the method used to extract the tears from the absorbent materials, that is, centrifugation or elution with a solvent. Doxycycline was selected as the test drug because it has been shown to reach the tear compartment in several species,^11–13^ it has broad-spectrum antimicrobial activity, and multiple beneficial properties such as inhibition of matrix metalloproteinases, anti-inflammation, immunomodulation, and antiangiogenesis.^23^

## Methods

### In vivo evaluation: determination of serum and tear levels

#### Animals

Three dogs and 3 cats were enrolled in the study; all confirmed to be ophthalmoscopically healthy by slit-lamp examination, indirect funduscopy, tonometry, Schirmer tear test, and fluorescein staining. Dogs comprised of 1 neutered male Chihuahua, 1 spayed female Great Pyrenees, and 1 spayed female Greyhound, with a mean ± standard deviation (SD) (range) age of 5.7 ± 3.5 years (2–9 years) and body weight of 19.3 ± 13.0 kg (6–32 kg). Cats were all Domestic Shorthair breed, comprised of 2 neutered male and 1 spayed female, with a mean ± SD (range) age of 3.7 ± 2.3 years (1–5 years) and body weight of 5.8 ± 1.4 kg (5–7.5 kg). An informed consent was obtained from all owners. The study was approved by the Institutional Animal Care and Use Committee of Iowa State University, and adhered to the Association for Research in Vision and Ophthalmology (ARVO) statement for the Use of Animals in Ophthalmic and Vision Research.

#### Drug administration and sample collection

**FIG. 1.**
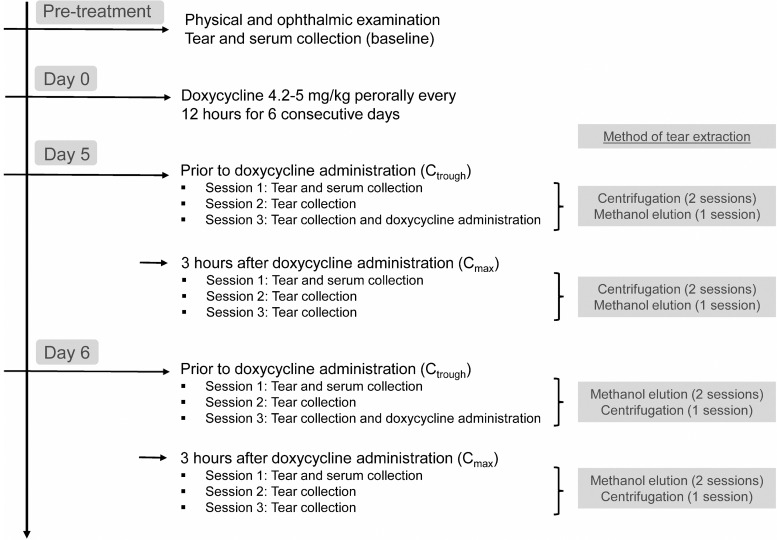
Diagram of the study design used to assess 3 healthy dogs and 3 healthy cats who were administered oral doxycycline (4.2–5.0 mg/kg every 12 h for 6 days). At both day 5 and 6, serum and tear samples were collected to capture trough (*C*_trough_) and maximal (*C*_max_) concentrations. Tears were collected 3 times at each session, separated 10 min apart, and fluid was extracted from the absorbent material with either centrifugation or methanol elution.

Doxycycline hyclate was administered orally at a dose of 4.2–5 mg/kg every 12 h for 6 consecutive days. On both the fifth and sixth day of drug administration, all subjects had blood and tear fluid sampled to capture doxycycline trough concentration (*C*_trough_; before morning drug administration) and maximal concentration (*C*_max_; 3 h after morning drug administration), as described in Fig. 1. The duration of drug administration preceding the first sample collection was selected to ensure steady-state doxycycline plasma concentrations were reached, as the plasma half-life was reported to be 10.36 h in dogs^24^ and 4.24 h in cats.^12^

Blood was collected once from each subject at each session (*C*_trough_ and *C*_max_ at day 5 and 6): the 2–3 mL blood sample retrieved by peripheral venipuncture was placed in a plain red top tube (containing no anticoagulant), centrifuged at 1,240 *g* for 10 min, and the extracted serum was stored at −80°C until analysis.

Tear fluid was collected 3 times from each subject at each session (*C*_trough_ and *C*_max_ at day 5 and 6), allowing 10 min between successive sampling in the same eye to permit return to basal tearing status.^22^ For each subject, the right and left eyes were randomized (Excel 2016; Microsoft, Redmond, WA) to receive either Schirmer strips (Eye Care Product Manufacturing, LLC, Tucson, AZ) or polyvinyl acetal (PVA) sponges (Ultracell^®^ Eye Wick; Beaver-Visitec International, Inc., Waltham, MA), and this selection was kept consistent over the several sessions. Collection with Schirmer strips was performed by placing the filter paper in the lower conjunctival fornix until the strip was wet at the 20-mm mark. Collection with PVA sponges was performed as previously described^22^—briefly, a 4 × 10 mm strip of PVA sponge was inserted into the lower conjunctival fornix using a disposable plastic tweezer (Evident Inc., Union Hall, VA), and was left in place for 60 s. Once soaked with tears, extraction of tear fluid from these absorbent materials was performed using 1 of 2 methods:

(1) Centrifugation: The wetted Schirmer strips and PVA sponges were placed in separate 0.2-mL Eppendorf tubes that were previously punctured at their bottom with an 18-gauge needle. Tears were then eluted through the drainage hole into 1.5-mL Eppendorf tubes by centrifugation for 1 min at 3,884 *g* (VWR^®^ Mini Centrifuge; VWR International, Radnor, PA).
(2) Methanol elution: The wetted Schirmer strips and PVA sponges were placed in separate1.5-mL Eppendorf tubes. Methanol (HPLC grade; Fisher Scientific, Raleigh, NC) was then added to each tube to immerse the entire absorbent material (1.5 mL for Schirmer strips, 1 mL for PVA sponges).

During each time point, 2 of the 3 tear samples collected from each eye were processed using the same extraction method (centrifugation on day 5, methanol elution on day 6; Fig. 1) so that intrasession variability could be assessed (see “Data Analysis” section).

Assuming tear fluid density equaled 1 g/mL,^25^ tear volume absorbed was calculated as the difference of the post- and precollection weight of the tube containing the Schirmer strip or PVA sponge (before centrifugation or methanol elution). Weights were determined to the nearest 0.001 *g* (Gemini-20; American Weight Scales, Inc., Norcross, GA). Samples were then placed in a −80°C freezer until analysis.

### In vitro evaluation: determination of doxycycline recovery from absorbent materials

Doxycycline hyclate (Sigma-Aldrich ≥98% HPLC; Sigma-Aldrich, St Louis, MO) was prepared as solutions of 100 and 1,000 ng/mL (representative of low and high tear concentrations obtained in the present *in vivo* experiment) in a buffer made of 50% acetonitrile (HPLC grade; Fisher Scientific, Raleigh, NC), 50% water (HPLC grade; Fisher Scientific, Raleigh, NC), and 0.1% formic acid. Twenty Schirmer strips (wet until the 20-mm mark was reached) and 20 PVA sponges (spiked with 25 μL, representative of the median volume absorbed by these sponges in the present *in vivo* experiment) were used for each doxycycline concentration, 10 of which underwent extraction with centrifugation while the remaining 10 underwent elution with methanol, following the same protocol as the *in vivo* experiment. Samples were then stored at −80°C until analysis.

### Analytical methods

#### Sample preparation

Serum and tear samples were thawed and vortexed gently for 1 min (Vortexer 120V; Fisher Scientific, Hampton, NH). Tear samples obtained by methanol elution were centrifuged for 6 min at 10,000 *g* (Labnet International, Edison, NJ), and the supernatants (0.5 mL for PVA samples and 1.0 mL for Schirmer samples) were transferred to LC-MS vials. The samples were dried with a nitrogen evaporator for 20 min, then resuspended in 200 μL of buffer (1:1 acetonitrile:water with 0.1% formic acid). For tears obtained by centrifugation and for serum samples, 7–10 and 100 μL of each sample type, respectively, were transferred to 1.5-mL microcentrifuge tubes and mixed with 200 or 900 μL of buffer (1:1 acetonitrile:water with 0.1% formic acid), respectively. These samples were then vortexed for 30 s, left on ice for 10 min, and vortexed for another 30 s before being sonicated in an ice water bath for 10 min. Following centrifugation for 7 min at 13,000 *g*, the supernatant from each sample was transferred to a 0.2 μL spin filter and centrifuged again for 7 min at 13,000 *g*.

#### Standard solutions

The following concentrations of doxycycline hyclate were prepared in a buffer of 1:1 acetonitrile:water with 0.1% formic acid: 1.0, 5.0, 10, 20, 50, 100, 625, 1,000, 1,250, 2,500, and 5,000 ng/mL. Tear calibration curve was made by analyzing doxycycline solutions of 1, 5, 10, 20, 50, and 100 ng/mL that were each mixed with 10 μL of blank canine and feline tears. Serum calibration curve was made by analyzing doxycycline solutions of 625, 1,250, 2,500, and 5,000 ng/mL that were each mixed with 30 μL of blank canine and feline serum. A linear correlation was observed within the range of concentrations considered for tears (*R*^2^ = 0.989) and for serum (*R*^2^ = 0.996).

#### Liquid chromatography/mass spectrometry

An Agilent 6540 Ultra High-Definition Accurate-Mass Q-TOF liquid chromatography/mass spectrometry (LC/MS) system was used for sample analysis (Agilent Technologies, San Jose, CA). LC separations were performed with an Agilent Technologies' 1290 Infinity Binary Pump UHPLC instrument equipped with an Agilent Technologies' Eclipse C18 1.8 μm 2.1 mm × 50 mm analytical column that was coupled to an Agilent Technologies' 6540 UHD Accurate-Mass Q-TOF mass spectrometer. A volume of 15 μL of each sample was injected into the device, with an initial buffer condition of 100% water progressing to 100% acetonitrile with 0.1% formic acid over the 10-min run period. Chromatography was carried out at 40°C with a flow rate of 0.6 mL/min. Doxycycline was detected as [M+H]^+^ ions using electrospray ionization in positive mode, while operating in high-resolution (4 Gz) mode with a scan range from m/z 100 to m/z 1,700. Doxycycline [M+H]^+^ ions were plotted as an extracted ion chromatogram using a 10 ppm extraction window centered around m/z 445.16, and the observed retention time for doxycycline was 3 min.

Analyses of blank tear fluid spiked with 2 doxycycline standard solution (100 and 1,000 ng/mL) were performed to serve as control for the *in vitro* study. For the *in vivo* experiments, the concentrations of doxycycline in the tear fluid and serum were estimated in ng/mL on the basis of peak area from the tears and serum calibration curves, respectively, taking into account the various dilutions performed during the sample preparation.

### Data analysis

Normality of the data was assessed with the Shapiro–Wilk test. For normally distributed data, parametric tests were used for analysis and results were expressed as mean ± SD. Non-normally distributed data were analyzed with nonparametric statistics and results were expressed as median and 95% range (2.5th percentile–97.5th percentile). Comparison of serum doxycycline levels was performed with the unpaired *t*-test (dogs vs. cats) and the Mann–Whitney rank test (*C*_trough_ vs. *C*_max_). Comparison of tear doxycycline levels between dogs versus cats, and between *C*_trough_ versus *C*_max_ were performed with the Kruskal–Wallis test followed by Dunn's pairwise comparison, taking into account the following 4 collection/extraction methods: (1) Schirmer strip/centrifugation, (2) Schirmer strip/methanol elution, (3) PVA sponge/centrifugation, and (4) PVA sponge/methanol elution. When no statistical differences were noted, data from both species as well as both trough and maximal concentrations were combined for future analysis, described thereafter as “serum doxycycline concentration” and “tear doxycycline concentration.” Differences in the doxycycline concentrations obtained *in vivo* and *in vitro* with each of the aforementioned group were evaluated with the Kruskal–Wallis test followed by Dunn's pairwise comparison.

#### Correlations

Spearman's rank correlation tests were used to assess correlations between the following parameters: (1) tear doxycycline concentration versus tear flow rate (defined as the volume absorbed by the absorbent material per minute), and (2) serum doxycycline concentration versus tear doxycycline concentration obtained by each of the 4 collection/extraction methods.

#### Intrasession variability

The variability of the tear doxycycline concentrations obtained by the same method during the same session was evaluated with the coefficient of variation (CV), expressed in %, defined as the ratio of the SD by the mean values of doxycycline concentrations. Differences in CV% between Schirmer strips and PVA sponges were evaluated with the Mann–Whitney test, whereas differences in CV% among the 4 collection/extraction methods were assessed with the Kruskal–Wallis test.

## Results

Data were non-normally distributed (*P* < 0.05), except for serum doxycycline concentrations (*P* ≥ 0.354) and tear volume absorbed (*P* ≥ 0.118).

### In vivo evaluation: determination of serum and tear levels

Serum concentrations of doxycycline were not statistically different between trough and maximal measurements in both species (*P* ≥ 0.650). The drug could be quantified in all sera samples collected: mean ± SD (range) concentrations were 1301.8 ± 602.0 ng/mL (469.1–2356.5 ng/mL) in dogs and 1531.6 ± 804.6 ng/mL (329.5–3208.4 ng/mL) in cats, a difference that was not statistically significant (*P* = 0.437). Mean ± SD (range, CV%) tear volume absorbed was 17.6 ± 2.3 μL (13–22 μL, 13%) for Schirmer strips and 27.6 ± 17.7 μL (3–70 μL; 64%) for PVA sponges. Tear concentrations of doxycycline were below the limit of quantification (<1 ng/mL) in 29/144 samples collected, a finding mostly noted in cats and methanol-eluted tears (Appendix 1). Median (95% range) tear levels were 182.0 ng/mL (45.4–632.0 ng/mL) in dogs and 172.0 ng/mL (45.1–900.7 ng/mL) in cats, a difference that was not statistically significant (*P* = 0.865).

Tear concentrations were not statistically different between trough and maximal concentrations in both species and in both collection methods (*P* = 1.000; Table 1). In tear samples collected with Schirmer strips, centrifugation extraction achieved greater doxycycline levels than methanol elution for both *C*_trough_ (*P* = 0.002) and *C*_max_ (*P* < 0.001; Table 1). In tear samples collected with PVA sponges, doxycycline levels were greater with centrifugation extraction for *C*_max_ (*P* = 0.025), but not *C*_trough_ (*P* = 0.121; Table 1).

**Table 1.** Median ± Standard Error (95% Range) Trough and Maximal Concentrations of Doxycycline in Tear Fluid of 3 Healthy Dogs and 3 Healthy Cats Receiving 4.2–5 mg/kg Doxycycline Per orally for 6 Consecutive Days

	*Schirmer strips*		*PVA sponges*	
In vivo *Doxycycline concentration*	*Centrifugation*	*Methanol elution*	*Centrifugation*	*Methanol elution*
*C*_trough_ (ng/mL)	279.6 ± 24.2 (143.3–458.2)^*^	63 ± 57.9 (44.8–543.7)	176 ± 59.2 (119.3–831.8)	65.4 ± 12.7 (33.7–138.8)
*C*_max_ (ng/mL)	441.1 ± 79.2 (308.8–1388.1)^*^	87 ± 53.9 (53.9–156.6)	278 ± 26.1 (186.4–552.5)^*^	96.8 ± 57.2 (38.7–650.7)

Tears were collected *in vivo* with either Schirmer strips or PVA sponges, and fluid was subsequently extracted by centrifugation or methanol elution. For the same absorbent material, significant differences between centrifugation versus methanol are noted with an asterisk (^*^).

PVA, polyvinyl acetal.

#### Correlations

**FIG. 2.**
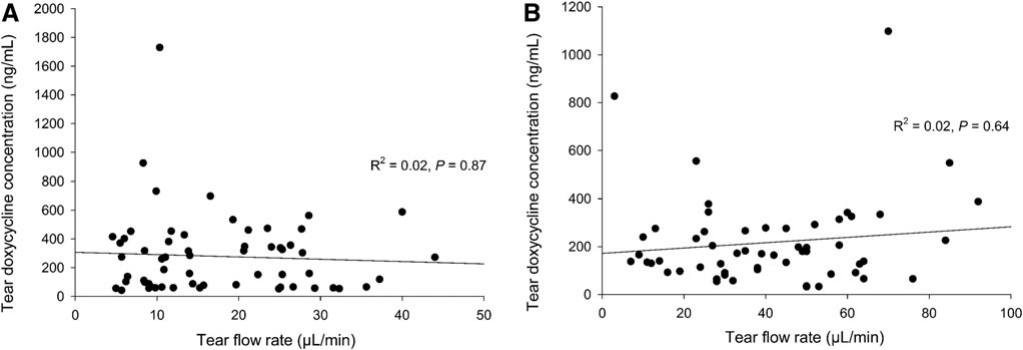
Spearman's rank correlation test for tear doxycycline concentrations and tear flow rate. No significant associations were found for either Schirmer strips **(A)** or PVA sponges **(B)**. PVA, polyvinyl acetal.

Concentrations of doxycycline were not correlated between serum and tear fluid for any collection/extraction method (*P* ≥ 0.221), except for Schirmer strips followed by centrifugation (*r* = 0.424, *P* = 0.013). Furthermore, no significant correlation was noted between tear flow rate and tear doxycycline concentrations for either Schirmer strips (*P* = 0.87; Fig. 2A) or PVA sponges (*P* = 0.640; Fig. 2B).

#### Intrasession variability

**FIG. 3.**
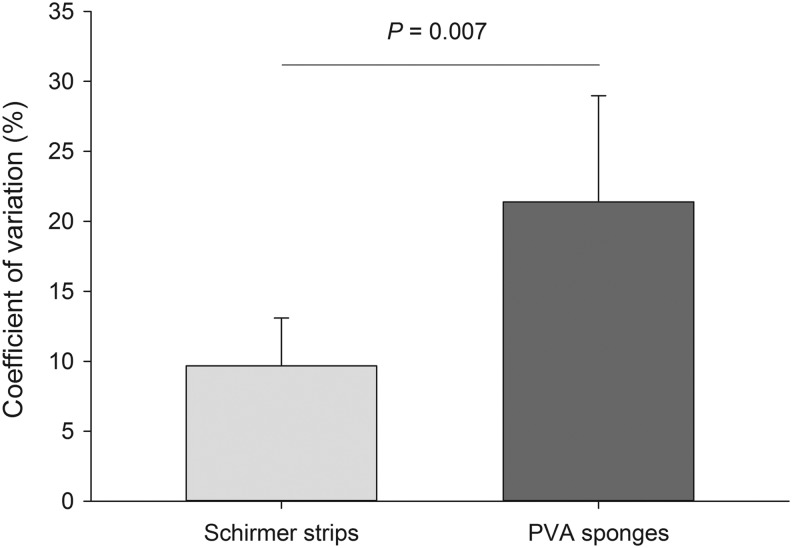
Bar chart depicting median + standard error of the coefficient of variation in doxycycline concentrations of tears collected *in vivo* with Schirmer strips or PVA sponges. A significant difference was noted between both collection methods (*P* = 0.007). PVA, polyvinyl acetal.

Median ± standard error (95% range) CV% of tear samples collected with Schirmer strips (9.7% ± 3.4%, 1.1%–46.6%) was significantly lower (*P* = 0.007) compared with samples collected with PVA sponges (21.4% ± 7.6%; 6.7%–93.0%; Fig. 3). When considering the subsequent extraction method, CV% was confirmed to be lower for Schirmer strips [centrifugation: 8.5% ± 5.0% (0.8%–47.6%); methanol elution: 14.3% ± 3.8% (2.8%–25.3%)] compared with PVA sponges [centrifugation: 21.4% ± 8.7% (8.5%–92.5%); methanol elution: 34.2% ± 17.6% (8.0%–76.3%)], although differences among these 4 groups were not statistically significant (*P* ≥ 0.054).

### In vitro evaluation: determination of doxycycline recovery from absorbent materials

Results of the *in vitro* experiment are summarized in Table 2. There were no significant differences (*P* ≥ 0.210) in the recovery of doxycycline between Schirmer strips and PVA sponges for either extraction methods (centrifugation or methanol-elution) or doxycycline solutions (100 and 1,000 ng/mL). Median CV% of doxycycline quantitation was overall similar between Schirmer strips and PVA sponges for the 100 ng/mL solution (35.7% and 32.1%, respectively) and the 1,000 ng/mL solution (19.4% and 27.2%, respectively). The recovery of doxycycline from the absorbent materials was often greater with 100 ng/mL versus 1,000 ng/mL of doxycycline solution, and this difference was statistically significant for centrifuged PVA sponges (*P* < 0.001).

**Table 2.** Median ± Standard Error (95% Range) Percentage Recovery of Doxycycline *In Vitro* from Schirmer Strips and PVA Sponges Spiked with Either 100 or 1,000 ng/mL Doxycycline Solutions, Then Processed with Either Centrifugation or Methanol Elution

	*Schirmer strips*		*PVA sponges*	
In vitro *Doxycycline solution*	*Centrifugation*	*Methanol elution*	*Centrifugation*	*Methanol elution*
100 ng/mL	60.7 ± 13.2 (32.1–100.8)	52.3 ± 3.4 (46.3–64.5)	75.0 ± 1.9 (67.4–77.0)^*^	38.4 ± 2.0 (35.2–44.1)
1,000 ng/mL	31.8 ± 3.0 (22.6–38.5)	40.2 ± 1.0 (39.4–43.4)	24.8 ± 1.1 (22.9–27.5)	40.0 ± 2.1 (37.1–45.6)

For the same absorbent material and the same extraction method, significant differences between the recovery from 100 ng/mL versus 1,000 ng/mL solutions are noted with an asterisk (^*^).

PVA, polyvinyl acetal.

## Discussion

Precise quantification of tear fluid concentrations following systemic therapy is critical for many reasons. First, systemic medications can be used to treat various ocular surface diseases, and accurate tear concentrations are necessary to determine dose and frequency of administration. Second, lacrimal fluids can be used to correlate drug concentrations with ocular adverse effects of systemic drugs, such as conjunctivitis from nonsteroidal anti-inflammatories^26^ or drug-induced corneal epithelial changes.^27^ Third, tears have been shown to be the best practical indicator of the unbound fraction of various drugs, such as phenobarbital^28^ and theophylline,^29^ and therefore represent an excellent alternative to invasive blood or cerebrospinal fluid collection.

After oral administration, doxycycline reached the tear compartment at concentrations of 45.1–900.7 ng/mL in cats and 45.4–632.0 ng/mL in dogs. In fact, these values may represent underestimations of the “true” concentrations, as our preliminary *in vitro* data showed an incomplete recovery of doxycycline from the absorbent materials with the extraction protocols used in the present study. Furthermore, the analytical method used in the present study did not use an isotopically labeled internal standard of doxycycline, a limitation that likely affected the accuracy of the measurements from the LC/MS assay.^30^ Thus, the range of tear concentrations in dogs and cats needs to be substantiated by additional studies with a larger population size. Regardless of these limitations, these measured concentrations are up to 9-fold and 1,000-fold greater than those previously reported in cats and dogs, respectively.^12,13^ Because serum doxycycline concentrations were overall consistent with data published in previous canine and feline studies using similar doses of oral doxycycline,^12,31^ the large discrepancy in concentrations measured in the lacrimal fluid could be imputed, at least partially, to the different methodologies used for tear collection and extraction.

The average tear-to-serum ratio of doxycycline concentrations was 16% in dogs and 12% in cats, somewhat similar to penciclovir (20%)^2^ but lower than pradofloxacin in cats (36–1,818%).^12^ These differences in lacrimal distribution might be due to physicochemical properties inherent to each drug such as lipophilicity, protein binding, molecular mass, and p*K*_a_.^10^ Of note, the tear-to-serum ratio was greater in tear samples collected with Schirmer strips and extracted with centrifugation (27%) compared with other methods (5%–16%). This, combined with the steady volumes of tear fluid obtained with Schirmer strips at each collection time (20-mm mark wetting), may partly explain the positive correlation noted for this method between serum and tear concentrations of doxycycline. Furthermore, it is important to note that our work and the aforementioned studies were performed in healthy eyes. Breakdown of the blood–ocular barrier, as seen with conjunctivitis-induced vessel fragility, may increase the tear-to-serum ratio by allowing direct passage of drug from the serum to the tear fluid.^1^ In humans with allergic conjunctivitis, oral administration of cetirizine achieved tear concentrations nearly identical to serum concentrations after 60 min, indicating that a pharmacokinetic equilibrium has been reached relatively quickly between the 2 compartments.^1^ In humans with trachomatous conjunctivitis, administration of minocycline achieved sustained tear concentrations for 48 h following a single oral dose.^32^ Future studies should investigate the impact of conjunctivitis on lacrimal drug levels.

In the present study, doxycycline tear concentrations were not correlated with tear flow rate in dogs and cats. The stable concentration profile of tear doxycycline over a wide range of tear flow rates suggest that the drug secretion into the tear compartment increases with the rate of lacrimal stimulation. This is similar to “regulated” lacrimal proteins, such as lysozyme and lactoferrin,^33^ but is in contrast to “constitutive” and “serum-derived” tear compounds, such as IgA and IgG, respectively, for which the tear concentrations decrease with increasing stimulus due to dilution effect.^33^ Additionally, it is plausible that doxycycline accumulates in the lacrimal gland after days of oral administration, similar to cyclosporine A,^18^ thus explaining the pattern of concentrations observed in the present study.

Importantly, our study found that doxycycline tear concentrations were more repeatable when tear collection was performed with Schirmer strips rather than PVA sponges. Schirmer strips are thinner than PVA sponges (0.22 mm vs. 1 mm) and this may help extract the drug from the absorbent material in a more consistent way, thus providing a possible explanation for the lower variability of doxycycline in tear samples. Further, differences in water affinity between Schirmer strips and PVA sponges,^22^ combined with different physical properties, such as porosity, pore size, and permeability,^34^ may explain the (nonsignificant) discrepancy noted herein in the quantity of doxycycline extracted from each collection device.

Of note, Schirmer strips allow for standardization of the tear volume absorbed at each session by controlling the mm-mark of wetness (intersession differences in VA ≤4 μL), whereas PVA sponges yield more variable tear volumes despite controlling for the collection duration (intersession differences in VA ≤37 μL). This variability is likely due to reflex tearing, and although it did not impact doxycycline concentrations in the present study (no correlation between tear flow rate and doxycycline concentrations), it may participate in data variability when tear sampling is not performed at steady-state plasma levels. Indeed, a recent study on minocycline collected tear fluid in ponies with ophthalmic sponges, and the authors noted large discrepancies in lacrimal drug concentrations between one subject to another.^35^ Therefore, we believe that Schirmer strips represent a better option for lacrimal doxycycline quantification, offering a good compromise between collection of a sufficient amount of tears (for allowing bioanalytical quantitation) and patient tolerance (for the respect of lacrimal physiology). However, this finding should be verified for other drugs as results may be affected by diverse compound characteristics (eg, size, lipophilicity, and protein binding). Also, given the several *in vivo* samples that were below the quantification limit and our preliminary *in vitro* data showing suboptimal drug recovery, tear collection with Schirmer strips has to be optimized for it to become the standard collection method for pharmacokinetic studies. Indeed, postcollection processing of Schirmer strips has been shown to impact the lacrimal protein content,^36^ and the same is likely to be true for drug levels. The following are a few suggestions that can be investigated in future studies:

(1) Adjust the volume absorbed by the Schirmer strip to collect only the basal lacrimal lake (6.2–7.0 μL in humans,^37^ unknown in dogs and cats), so that any dilution effect from reflex tearing is reduced.^19^ If so, extraction of the tears with solvent elution would be preferable, as the amount of fluid recovered by centrifugation of such low volumes is often negligible (<5 μL; L. Sebbag, unpublished observations).
(2) Improve the extraction of drug by cutting the Schirmer strip in small pieces,^13^ allowing sufficient contact time between the solvent and the Schirmer strip,^36^ and using ultrasonic agitation to facilitate diffusion.^12,20^
(3) Establish the analytical calibration curve by spiking Schirmer strips with known concentrations of the drug of interest, and processing these standards the same way as the biological samples.^38^ This approach may help account for the potential adsorptive properties of the Schirmer strips for a wide range of drug concentrations.

## Appendix

**Appendix A1.** Number of Tear Samples in Dogs and Cats for Which Doxycycline Measurement Was Below Limit of Quantification, Detailing the Collection Device and Extraction Method Used as Well as the Associated Volume of Tears Absorbed

	*Schirmer strips*		*PVA sponges*	
	*Centrifugation*	*Methanol elution*	*Centrifugation*	*Methanol elution*
Dogs				
Number of samples BLQ	0/18	3/18	0/18	4/18
Mean volume absorbed (μL)	18.3	18.9	40.3	19.1
Cats				
Number of samples BLQ	2/18	7/18	3/18	9/18
Mean volume absorbed (μL)	16.1	16.6	26.0	18.1

BLQ, below limit of quantification; PVA, polyvinyl acetal.
